# Screening, Evaluation, and Early Management of Acute Aortic Dissection in the ED

**DOI:** 10.2174/157340312801784970

**Published:** 2012-05

**Authors:** Reuben J Strayer, Peter L Shearer, Luke K Hermann

**Affiliations:** Mount Sinai School of Medicine, One Gustave L Levy Place Box 1149, New York, NY 10029

**Keywords:** Aortic aneurysm/diagnosis, aortic aneurysm/mortality, aortic aneurysm/therapy, d-dimer, echocardiography, guidelines, mortality, risk factors.

## Abstract

Acute aortic dissection (AAD) is a rare and lethal disease with presenting signs and symptoms that can often be seen with other high risk conditions; diagnosis is therefore often delayed or missed. Pain is present in up to 90% of cases and is typically severe at onset. Many patients present with acute on chronic hypertension, but hypotension is an ominous sign, often reflecting hemorrhage or cardiac tamponade. The chest x-ray can be normal in 10-20% of patients with AAD, and though transthoracic echocardiography is useful if suggestive findings are seen, and should be used to identify pericardial effusion, TTE cannot be used to exclude AAD. Transesophageal echocardiography, however, reliably confirms or excludes the diagnosis, where such equipment and expertise is available. CT scan with IV contrast is the most common imaging modality used to diagnose and classify AAD, and MRI can be used in patients in whom the use of CT or IV contrast is undesirable. Recent specialty guidelines have helped define high-risk features and a diagnostic pathway that can be used the emergency department setting. Initial management of diagnosed or highly suspected acute aortic dissection focuses on pain control, heart rate and then blood pressure management, and immediate surgical consultation.

## INTRODUCTION

Emergency physicians are especially challenged by diseases that are dangerous, require urgent treatment, and concealed from recognition. No diagnosis better exemplifies these characteristics than aortic dissection, and while the diagnosis is emphasized in Emergency Medicine training and continuing education, aortic dissection is not diagnosed on its initial presentation in 15-43% of cases [1,2]; in fact, one expert claims that "difficulty in diagnosis, delayed diagnosis or failure to diagnose are so common as to approach the norm for this disease, even in the best hands..." [3] Furthermore, patients with aortic dissection are often relatively young and healthy; the diagnostic edge could therefore not be sharper. When the condition is identified, therapy is driven by a set of key principles and plagued by several management pitfalls. A recently published multidisciplinary guideline offers both diagnostic and therapeutic pathways to assist clinicians caring for patients with suspected or confirmed aortic dissection.

## EPIDEMIOLOGY

Aortic dissection is an uncommon disease, with prevalence estimates ranging from 5,000 to 10,000 cases in the United States per year. [4,5] Untreated, aortic dissection carries a devastating mortality of 40% on presentation and an additional 1% rate of death per hour, to a 1 year mortality of 90%. [6] That the diagnosis is so often delayed in a disease known to be so lethal reflects the great diagnostic challenge aortic dissection poses to emergency clinicians.

Approximately 1 in 10,000 ED patients will have aortic dissection, a number so small that emergency providers may only see several cases in their career. Only one quarter of patients with aortic dissection present with a combination of classic features (pain of sudden onset or ripping/tearing quality, blood pressure differential, and widened mediastinum on chest radiograph); 1 in 25 patients diagnosed with aortic dissection has *none* of the classic features. [6] Furthermore, aortic dissection can cause myriad symptoms localizing to any organ system or body part, and each of these symptoms can be explained by more common conditions–often by more common *dangerous* conditions that quite reasonably establish the focus of care but ultimately turn out to be distractors.

Emergency clinicians are thus confronted with innumerable patients whose symptoms *could* be caused by aortic dissection but almost certainly *are not;* aortic dissection could therefore be said to represent not just a needle in a haystack, but a needle disguised as a blade of hay in a haystack. Physicians evaluating patients whose symptoms may be caused by aortic dissection must therefore understand the clinically relevant risk factors and clinical manifestations of this condition and develop a risk stratification strategy that catches as many patients with the disease as possible without overusing advanced imaging studies. 

## PATHOPHYSIOLOGY

Aortic dissection occurs when the innermost layer of the aortic vessel wall is torn, creating a false lumen which transmits a longitudinal column of blood. It is sometimes referred to as a *dissecting aortic aneurysm*; however this term is discouraged as it is both inaccurate and conflates aortic dissection with aortic aneurysm, a distinct clinical entity. Aortic dissection is thought to result from the hydrostatic pressure accumulated as blood is pumped through the aorta, as well as movement of the aorta itself, with every cardiac cycle. Histologically, aortic dissection is associated with characteristic changes in the vessel wall known as *medial degeneration*. The former term, *cystic medial necrosis*, has fallen out of favor as the observed lesion demonstrates neither cysts nor necrosis.

Aortic dissection is more likely to occur in conditions that augment the pressure exerted by blood on the vessel wall, as well as conditions that weaken the vessel and accelerate medial degeneration. These conditions include hypertension, stimulant use (especially cocaine), pregnancy, weight-lifting or valsalva, pheochromocytoma, bicuspid aortic valve, aortic valve instrumentation or aortic surgery, syphilitic aortitis, large-vessel vasculitides, and congenital connective tissue disorders such as Marfan, Loeys-Dietz and Ehlers-Danlos syndromes.

The Stanford classification designates Type A dissections as lesions involving the ascending aorta, and Type B dissections confined to the descending aorta. Type A dissections are more common and much more dangerous, which drives differences in the therapeutic approach. Variants of aortic dissection include aortic intramural hemorrhage, which is a hematoma completely contained within the vessel wall, and penetrating aortic ulcer, a disruption in the vessel wall that usually leads not to dissection but aneurysm. These lesions are both treated similarly to aortic dissection. 

Aortic dissection causes morbidity and mortality by several mechanisms. Type A dissections can progress proximally to cause hemopericardium with tamponade as well as acute aortic valve regurgitation. Both types of dissections can breach the outer adventitial layer of the vessel, leading to free rupture into the chest or abdomen. Most sequellae of aortic dissection, however, result from the false lumen extending across ostia of branch arteries, leading to acute ischemia of potentially any organ in the body.

## CLINICAL FEATURES

Despite the rarity of the disease, good data are available on clinical features of aortic dissection, owing to The International Registry of Acute Aortic Dissection (IRAD). [7] Pain is present in 90% of cases and is more likely to be abrupt and most severe at onset than tearing or ripping. Pain location is a reflection of the site of the lesion and includes chest pain radiating to the neck, jaw or, classically, the back, thoracic or lumbar back pain, and abdominal pain. Constitutional symptoms are often marked and include nausea, diaphoresis, and, classically, extreme apprehension with a (justified) sense of impending doom. Patients with aortic dissection present with focal neurologic symptoms in 17% and syncope in 9% of cases. Though scenarios classically associated with aortic dissection such as migratory pain, chest pain with neurological deficits, and chest pain of sudden onset or with pulse deficit occur in only a minority of cases, their presence is strongly suggestive of the disease. 

Many patients with aortic dissection will present with acute on chronic hypertension, and aortic dissection is one of the cardinal hypertensive emergencies. Hypotension is ominous in the setting of aortic dissection as it often indicates either proximal extension with cardiac tamponade or free or contained rupture. Pseudohypotension–peripheral hypotension with central normotension–may be caused by dissection across the subclavian arteries. Subclavian or iliac artery embarrassment may also lead to the classic (though uncommon) finding of a pulse deficit or blood pressure differential across limbs. The murmur of aortic regurgitation, or signs of cardiac tamponade, may be present. If the dissection involves the left or (more commonly) right coronary artery, acute myocardial infarction and its attendant signs and symptoms can result. A variety of neurologic deficits including weakness or even coma may be caused by aortic dissection, depending on the cerebral branch arteries affected. Distal aortic dissection can cause ischemia to either kidney, lower extremity ischemia, as well as mesenteric ischemia and resulting abdominal pain, back pain, or diarrhea.

## DIAGNOSIS

Routine laboratory testing is not helpful for ruling in or ruling out aortic dissection. Preoperative studies are indicated when sufficient concern for the disease exists, or as soon as the diagnosis is confirmed.

The use of serum quantitative D-dimer testing has been proposed as a strategy to rule out aortic dissection. [8-12] Proponents of this approach suggest that blood in the false lumen activates the clotting cascade, generating fibrin degradation products detected by modern D-dimer assays with high sensitivity. Unfortunately, further work has demonstrated an unacceptably high false negative rate; in one study D-dimer was falsely negative in 9 of 113 confirmed aortic dissection cases. [13] A proposed explanation for false negative D-dimer assays is the occurrence of an aortic dissection variant featuring an anatomic thrombosed lumen which does not communicate with circulating blood, isolating the clot from detection by serum testing. [13,14] Furthermore, there is no evidence that D-dimer testing can be incorporated into a larger risk stratification strategy that would allow clinicians to sensitively exclude aortic dissection without greatly expanding the number of patients who receive advanced imaging studies. Given the experience with D-dimer testing to rule out pulmonary embolism–which has increased the number of advanced imaging studies ordered without increasing the number of pulmonary embolism diagnoses– [15] a comprehensive approach that accounts for false negatives and false positives should be validated before D-dimer testing is used routinely in the diagnosis of aortic dissection.

Plain chest radiography is indicated in all patients with chest pain of uncertain etiology, and whenever aortic dissection is considered. The chest xray is most useful when it provides an alternative explanation for the patient's symptoms. A variety of CXR abnormalities are associated with aortic dissection (Table **1**), the most important being mediastinal widening, which is present in more than half of cases. The absence of suggestive findings on chest xray makes aortic dissection less likely, however, 10-20% of patients with aortic dissection have a normal chest xray; therefore a negative study cannot exclude the disease and should not play a decisive role in the decision to pursue advanced imaging.

The electrocardiogram in aortic dissection may demonstrate changes associated with longstanding hypertension, as well as nonspecific ST-T wave changes; however the importance of ECG testing is to evaluate the differential diagnosis. The symptoms of aortic dissection overlap with the symptoms of myocardial ischemia, however a clinical challenge arises from the ability of aortic dissection to *cause *myocardial ischemia. When myocardial infarction complicates aortic dissection, treatment is directed at aortic dissection; furthermore, usual therapies for AMI may directly worsen outcomes of patients with aortic dissection. The clinician caring for a patient whose symptoms may be caused by either diagnosis must therefore have a strategy for managing their possible convergence, as discussed below.

Transthoracic echocardiography (TTE) poorly visualizes much of the aorta and is limited by patient, operator, and machine characteristics; aortic dissection cannot be excluded by TTE. However, point of care ultrasound by emergency physicians is recommended for all patients with suspected aortic dissection as well as all critically ill patients, and should be considered for all patients with chest or abdominal pain of uncertain etiology. In addition to evaluating alternative diagnoses, an intimal flap at either the aortic root or descending aorta may be distinguished by TTE and is diagnostic. TTE also reliably identifies complications of aortic dissection such as pericardial effusion and aortic regurgitation. [16-18]

Transesophageal echocardiography (TEE) is very accurate in both ruling in and ruling out aortic dissection and can be performed in a critical care area as resuscitative efforts are ongoing–a distinct advantage compared to CT and MRI, the two other definitive imaging modalities. However, TEE is uncommonly performed by emergency clinicians and not widely available on a consultative basis in many emergency departments. Invasive echocardiography may play a more prominent role in the emergency evaluation of aortic dissection if the technique sees broader application by emergency providers. [19]

Intravenous contrast-enhanced computed tomography reliably confirms and excludes aortic dissection and may elucidate alternative diagnoses, including pulmonary embolism and obstructive coronary artery disease. Contemporary CT scanning is rapid and widely available; CT is therefore the most common definitive imaging study used in patients with suspected aortic dissection. Beyond the concerns raised by moving a potentially critically ill patient to the radiology suite, drawbacks of CT include the risks of IV contrast and ionizing radiation.

Magnetic resonance imaging also accurately rules in and rules out aortic dissection, [20] and is free of contrast and radiation risk; however, limited availability and relatively long image acquisition times relegate MRI to a secondary imaging modality in most scenarios. MRI has a role in managing stable patients with an equivocal CT or TEE, or patients with known severe IV contrast allergy.

## EVALUATION

**Fig. (1):**
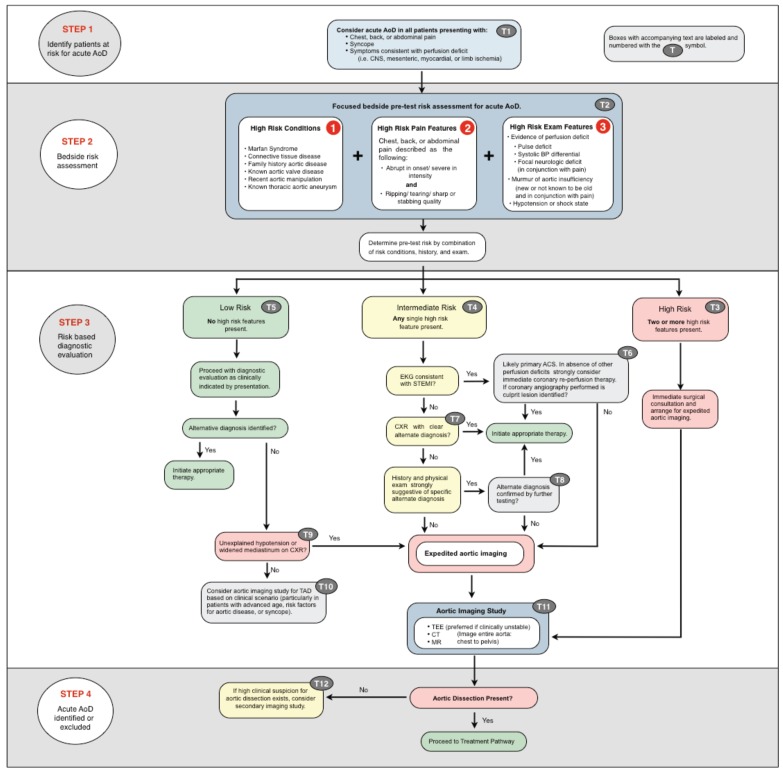
AoD Evaluation Pathway.

In April of 2010, the American College of Cardiology Foundation and American Heart Association published the first comprehensive, multidisciplinary guideline on the diagnosis and management of patients with thoracic aortic disease, including aortic dissection. [21] The authors present an *evaluation pathway* that specifically addresses what for emergency and primary care clinicians is the most difficult and crucial element of aortic dissection management: deciding which patients require advanced imaging to exclude the disease. (Fig. **1**) The pathway hinges on the assessment of pre-test probability of disease using specified high risk features from three risk categories: past medical history, history of present illness, and physical exam. All of these elements are collected on routine emergency assessments, with the exception of blood pressure measurement in both arms.

Patients with high risk features from more than one risk category are assumed to have aortic dissection until proven otherwise and should be managed accordingly. In this group of high risk patients with a negative definitive imaging study, a second imaging study should be strongly considered.

Patients with high risk feature(s) from only one risk category should have an electrocardiogram and chest xray performed promptly. If the electrocardiogram demonstrates evidence of acute myocardial infarction, management should be directed at AMI unless other strong evidence of aortic dissection is present. This recommendation is based on estimates of primary AMI occurring over one thousand times more frequently than AMI resulting from aortic dissection, [22] and the benefit of timely treatment of STEMI. For patients with one risk high risk feature and nondiagnostic ECG, expedited aortic imaging (CT, MRI, or TEE) is recommended for patients who do not have an alternative diagnosis identified on history, physical exam, or CXR.

Patients without high risk features only require aortic imaging if no alternative diagnosis is identified and the patient has unexplained hypotension or widened mediastinum on CXR.

The primary concern for emergency physicians managing confirmed or highly suspected aortic dissection is to arrange for immediate surgical consultation. Though Stanford B dissections may ultimately be managed non-surgically, all patients with aortic dissection should receive prompt surgical evaluation regardless of anatomic location. [21]

The goal of medical therapy in the normotensive or hypertensive patient with aortic dissection is to reduce the frequency and magnitude of force bloodflow exerts on the aortic wall. Analgesia is the first priority and is easily overlooked. Patients with aortic dissection may have severe pain and anxiety, both of which merit attending to in their own right but also produce a catecholamine response that directly undermines treatment objectives. Fortunately, unlike the underlying lesion, pain and anxiety are easily managed and intravenous opiates should be immediately and aggressively titrated to relief of pain as soon as dissection is diagnosed or strongly suspected.

The cornerstone of medical management is beta blockade, titrated to a heart rate of 60 beats per minute. Widely available agents well suited to this purpose include metoprolol and esmolol, with esmolol offering the benefit of minute-to-minute titration; this is particularly advantageous in aortic dissection patients who may experience dramatic swings in blood pressure as the lesion evolves and, for example, causes pericardial tamponade or acute aortic insufficiency. Labetalol is widely recommended and is an acceptable alternative; however labetalol tends to lower blood pressure more reliably than heart rate. [23,24] Patients with a strong contraindication to beta blockade should receive intravenous diltiazem or verapamil for rate control.

When beta blockade has achieved its goal heart rate, blood pressure is the next therapeutic target. If systolic blood pressure is greater than 120 mm Hg, an additional agent should be added to lower blood pressure with a goal of less than 120 mm Hg, ideally titrated to as low a blood pressure as end organs allow. Blood pressure should be measured in both arms and treatments directed at the highest reading. Nicardipine, a parental dihydropyridine calcium channel blocker, has emerged as the first line vasodilator infusion in many emergency departments and is recommended in this context; nicardipine may be replaced on formularies by its ultra-short acting congener, clevidipine, in the future. Nitroprusside is effective and classically used for aortic dissection, but is more difficult to manage and is associated with adverse effects such as cerebral blood vessel vasodilation [25] and cyanide or thiocyanate toxicity [26]. Fenoldopam, a peripheral dopamine agonist, and enalaprilat, an intravenous ACE inhibitor, are variously recommended as vasodilator therapies and are both acceptable choices but less easily titratable than the alternatives [25]. Phentolamine, hydralazine, and nifedipine should be avoided in aortic dissection if possible. Vasodilator agents should not be administered before chronotropic control is established with beta receptor or calcium channel blockade, as this may result in reflex tachycardia and an increase in aortic wall stress.

Medical therapies for patients with aortic dissection who are hypotensive are of minimal effect and limited to intravenous crystalloid and vasopressor support, pending surgical management. Pericardial tamponade is a common cause of hypotension in these cases; weak evidence suggests that pericardiocentesis should be avoided in patients not arrested or nearly arrested in favor of expeditious transfer to the operating theater. [27]

## CONCLUSION

Aortic dissection is an uncommon disease that often presents with varied and atypical findings suggestive of more frequently encountered conditions; it therefore poses an exceptional diagnostic challenge to emergency providers. Mortality associated with aortic dissection is significant at presentation and advances with every hour the lesion is left untreated. While the vast majority of patients who have symptoms possibly caused by aortic dissection will not have aortic dissection, key features of the disease including risk factors, pain characteristics, physical examination findings and signs on plain chest radiography allow clinicians to develop a rational approach to diagnostic testing supported by a recently-published multidisciplinary guideline. When the diagnosis is sufficiently likely to indicate definitive testing, the choice among advanced imaging modalities should be guided by institutional capabilities; ideally decided *a priori* with surgery colleagues. Patients with diagnosed or strongly suspected aortic dissection require expeditious surgical evaluation, aggressive analgesia, and treatment with rapid-acting, titratable agents to first lower heart rate and then blood pressure to specific targets.

## Figures and Tables

**Fig. (2):**
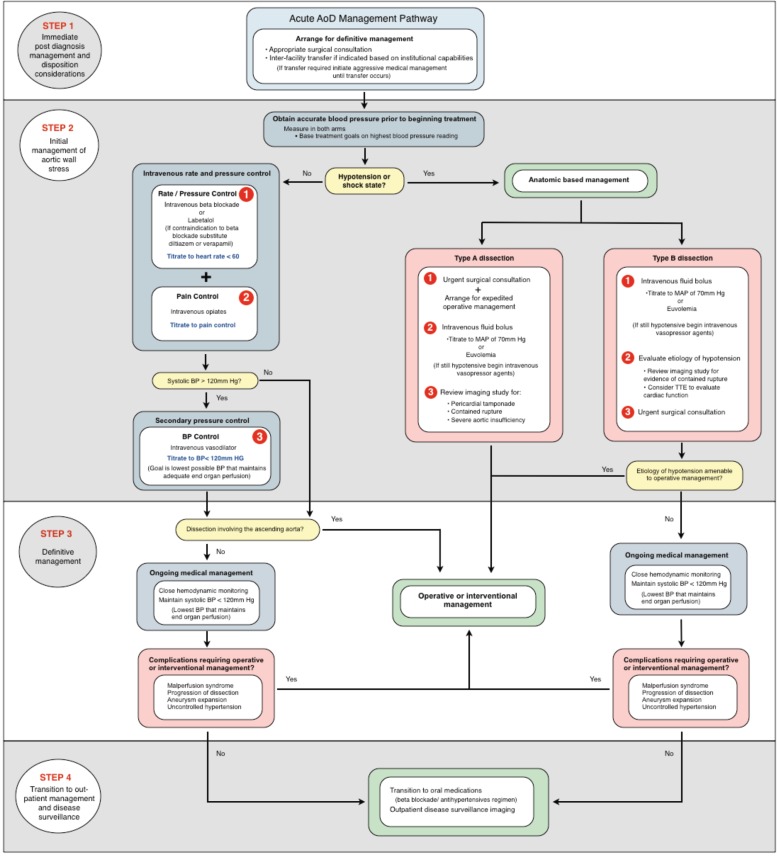
AoD Management Pathway.

**Table 1.** Signs of Aortic Dissection on Chest X-ray

Mediastinal widening
Disruption of normally distinct contour of aortic knob
Calcium sign - separation of intimal calcification from the vessel wall > 5 mm.
Double density appearance within aorta
Tracheal deviation to the right
Deviation of nasogastric tube to the right
